# 1-Benzoyl-4-thio­biuret

**DOI:** 10.1107/S1600536813019983

**Published:** 2013-07-27

**Authors:** Sung Kwon Kang

**Affiliations:** a Department of Chemistry, Chungnam National University, Daejeon 305-764, Republic of Korea

## Abstract

In the title compound (systematic name: {[(phenyl­formamido)­carbon­yl]amino}­methane­thio­amide), C_9_H_9_N_3_O_2_S, both benzoyl and terminal thio­urea fragments adopt *transoid* conformations with respect to the central carbonyl O atom. The benzoyl and thio­biuret groups are almost coplanar, making a dihedral angle of 4.40 (8)°. The mol­ecular structure is stabilized by two intra­molecular N—H⋯O hydrogen bonds. In the crystal, N—H⋯O and N—H⋯S hydrogen bonds link the mol­ecules into a tape running along [101].

## Related literature

For the structure and reactivity of thia­diazole derivatives, see: Cho, Ra *et al.* (1996 ▶); Cho, Cho *et al.* (1996 ▶). For the structure of a thio­biuret isomer, see: Kang *et al.* (2012 ▶).
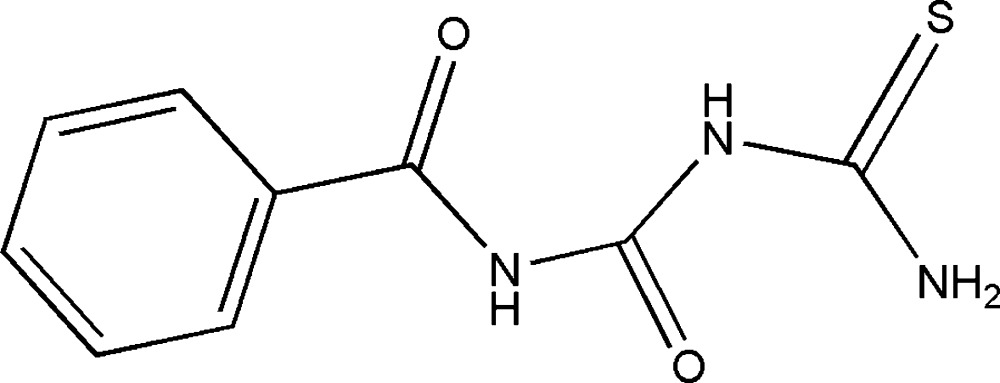


## Experimental

### 

#### Crystal data

C_9_H_9_N_3_O_2_S
*M* *_r_* = 223.25
Triclinic, 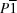

*a* = 5.6616 (1) Å
*b* = 7.8407 (2) Å
*c* = 11.7631 (3) Å
α = 97.169 (2)°
β = 94.992 (3)°
γ = 101.390 (2)°
*V* = 504.53 (2) Å^3^
*Z* = 2
Mo *K*α radiation
μ = 0.30 mm^−1^
*T* = 296 K
0.2 × 0.15 × 0.07 mm

#### Data collection

Bruker SMART CCD diffractometer
Absorption correction: multi-scan (*SADABS*; Bruker, 2002 ▶) *T*_min_ = 0.94, *T*_max_ = 0.97
17356 measured reflections
2516 independent reflections
1485 reflections with *I* > 2σ(*I*)
*R*_int_ = 0.055

#### Refinement

*R*[*F* ^2^ > 2σ(*F* ^2^)] = 0.037
*wR*(*F* ^2^) = 0.097
*S* = 0.87
2516 reflections
152 parameters
H atoms treated by a mixture of independent and constrained refinement
Δρ_max_ = 0.16 e Å^−3^
Δρ_min_ = −0.20 e Å^−3^

### 

Data collection: *SMART* (Bruker, 2002 ▶); cell refinement: *SAINT* (Bruker, 2002 ▶); data reduction: *SAINT*; program(s) used to solve structure: *SHELXS2013* (Sheldrick, 2013 ▶); program(s) used to refine structure: *SHELXL2013* (Sheldrick, 2013 ▶); molecular graphics: *ORTEP-3 for Windows* (Farrugia, 2012 ▶); software used to prepare material for publication: *WinGX* (Farrugia, 2012 ▶).

## Supplementary Material

Crystal structure: contains datablock(s) global, I. DOI: 10.1107/S1600536813019983/is5292sup1.cif

Structure factors: contains datablock(s) I. DOI: 10.1107/S1600536813019983/is5292Isup2.hkl

Supplementary material file. DOI: 10.1107/S1600536813019983/is5292Isup3.cml

Additional supplementary materials:  crystallographic information; 3D view; checkCIF report

## Figures and Tables

**Table 1:** Hydrogen-bond geometry (Å, °)

*D*—H⋯*A*	*D*—H	H⋯*A*	*D*⋯*A*	*D*—H⋯*A*
N9—H9⋯O11^i^	0.843 (18)	2.172 (18)	2.9891 (18)	163.3 (16)
N12—H12⋯O8	0.882 (19)	1.919 (19)	2.6171 (17)	135.0 (17)
N15—H15*A*⋯O11	0.91 (2)	1.99 (2)	2.684 (2)	132.0 (17)
N15—H15*B*⋯S14^ii^	0.85 (2)	2.58 (2)	3.4295 (17)	171.3 (17)

Symmetry codes: (i) 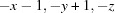; (ii) 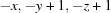.

**Fig. 1.**
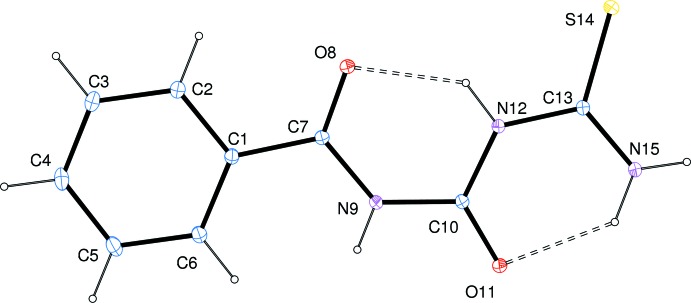
Molecular structure of the title compound, showing the atom-numbering scheme and 30% probability ellipsoids. Two intramolecular N—H···O hydrogen bonds are indicated by dashed lines.

**Fig. 2.**
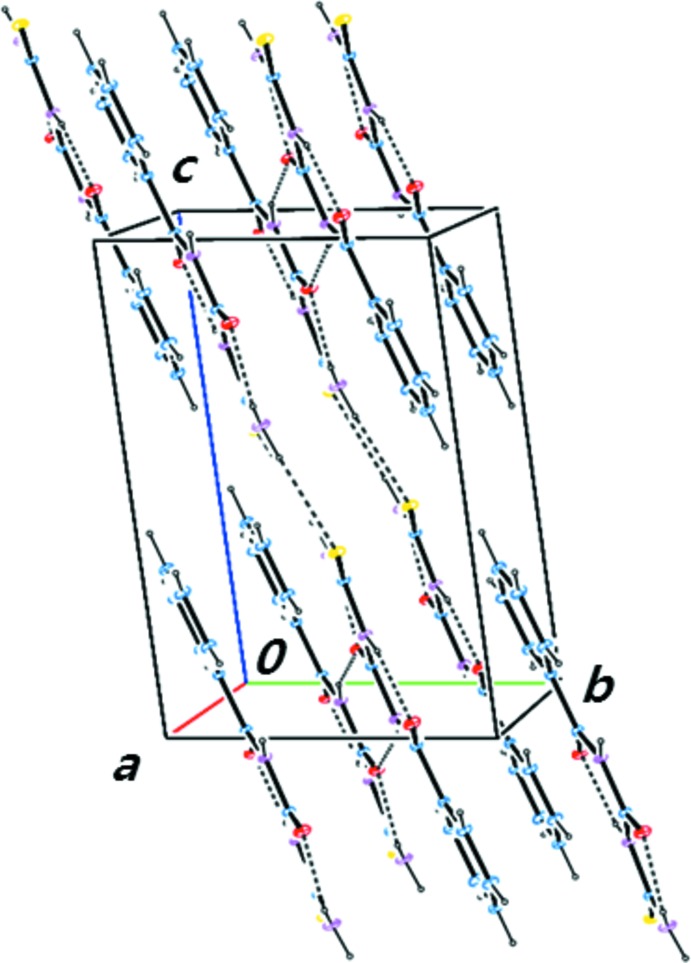
Part of the packing diagram of the title compound, showing a molecular tape formed by intermolecular N—H···O and N—H···S hydrogen bonds (dashed lines).

## supplementary crystallographic information

### Comment

5-Amino-2*H*-1,2,4-thiadiazol-3-one is the analog of cytosine. As an analog of cytosine, the tautomeric structure and reactivity of this compound have been examined (Cho, Ra *et al.*, 1996). Within the framework of our interest in the synthesis of novel potential anti-metabolites of nucleic acid components which would possess cytostatic activity, we have synthesized derivatives of 5-amino-3*H*-1,3,4-thiadiazol-2-one (Cho, Cho *et al.*, 1996). The title compound, 1-benzoyl-4-thiobiuret, is an isomer of 1-benzoyl-2-thiobiuret (Kang *et al.*, 2012). This compound is an intermediate for the formation of the thiobiuret which is a good starting material to make 5-amino-2*H*-1,2,4-thiadizolin-3-one *via* oxidative ring-closure reaction.

The dihedral angle between the benzoyl unit (C1–C7/O8) and thiobiuret group (N9/C10/O11/N12/C13/S14/N15) is 4.40 (8)°. Both carbonyl O8 and S14 atoms are positioned *anti* conformations with respect to the O11 atom (Fig. 1). The intramolecular O8···H12—N12 and O11···H15—N15 hydrogen bonds stabilize the molecule (Fig. 1 and Table 1). The intermolecular N—H···O and N—H···S hydrogen bonds link the molecules into a tape along the [101] direction (Fig. 2 and Table 1).

### Experimental

Benzoyl chloride (48 ml, 58.1 g, 0.41 mol) was added to warm solution of potassium thiocyanate (48.0 g, 0.49 mol) in acetone (400 ml). The solution became milky white and yellow when the addition had been completed. The mixture was stirred for 3.5 h at 50°C and left to cool to room temperature. The filtrate was heated to 55°C for 5 h with urea (24.0 g, 0.40 mol). And the resulting solution was cooled to room temperature and then placed in an ice bath for several hours. The cold mixture was filtered to give 1-benzoyl-4-thiobiuret as a bright yellow solid. Recrystallization from methyl alcohol afforded the yellow crystals suitable for X-ray diffraction.

### Refinement

H atoms of the NH and NH_2_ groups were located in a difference Fourier map and refined freely [refined N—H distances = 0.84 (2)–0.91 (2) Å]. Other H atoms were positioned geometrically and refined using a riding model, with C—H = 0.93 Å, and with *U*_iso_(H) = 1.2*U*_eq_(C).

### Crystal data

**Table:**

C_9_H_9_N_3_O_2_S	*Z* = 2
*M**_r_* = 223.25	*F*(000) = 232
Triclinic, *P*1	*D*_x_ = 1.47 Mg m^−^^3^
Hall symbol: -P 1	Mo *K*α radiation, λ = 0.71073 Å
*a* = 5.6616 (1) Å	Cell parameters from 3132 reflections
*b* = 7.8407 (2) Å	θ = 2.7–22.3°
*c* = 11.7631 (3) Å	µ = 0.30 mm^−^^1^
α = 97.169 (2)°	*T* = 296 K
β = 94.992 (3)°	Block, yellow
γ = 101.390 (2)°	0.2 × 0.15 × 0.07 mm
*V* = 504.53 (2) Å^3^	

### Data collection

**Table:**

Bruker SMART CCD diffractometer	1485 reflections with *I* > 2σ(*I*)
Radiation source: fine-focus sealed tube	*R*_int_ = 0.055
φ and ω scans	θ_max_ = 28.3°, θ_min_ = 1.8°
Absorption correction: multi-scan (*SADABS*; Bruker, 2002)	*h* = −7→7
*T*_min_ = 0.94, *T*_max_ = 0.97	*k* = −10→10
17356 measured reflections	*l* = −15→15
2516 independent reflections	

### Refinement

**Table:**

Refinement on *F*^2^	0 restraints
Least-squares matrix: full	Hydrogen site location: inferred from neighbouring sites
*R*[*F*^2^ > 2σ(*F*^2^)] = 0.037	H atoms treated by a mixture of independent and constrained refinement
*wR*(*F*^2^) = 0.097	*w* = 1/[σ^2^(*F*_o_^2^) + (0.052*P*)^2^] where *P* = (*F*_o_^2^ + 2*F*_c_^2^)/3
*S* = 0.87	(Δ/σ)_max_ < 0.001
2516 reflections	Δρ_max_ = 0.16 e Å^−^^3^
152 parameters	Δρ_min_ = −0.20 e Å^−^^3^

### Special details

**Table:**

Geometry. All e.s.d.'s (except the e.s.d. in the dihedral angle between two l.s. planes) are estimated using the full covariance matrix. The cell e.s.d.'s are taken into account individually in the estimation of e.s.d.'s in distances, angles and torsion angles; correlations between e.s.d.'s in cell parameters are only used when they are defined by crystal symmetry. An approximate (isotropic) treatment of cell e.s.d.'s is used for estimating e.s.d.'s involving l.s. planes.

### Fractional atomic coordinates and isotropic or equivalent isotropic displacement parameters (Å^2^)

**Table:**

	*x*	*y*	*z*	*U*_iso_*/*U*_eq_	
C1	0.0374 (3)	0.8224 (2)	−0.12506 (14)	0.0447 (4)	
C2	0.2488 (3)	0.9206 (2)	−0.15393 (16)	0.0597 (5)	
H2	0.3881	0.9477	−0.1014	0.072*	
C3	0.2552 (4)	0.9785 (3)	−0.25952 (19)	0.0729 (6)	
H3	0.398	1.0453	−0.2778	0.087*	
C4	0.0539 (4)	0.9383 (3)	−0.33677 (18)	0.0723 (6)	
H4	0.06	0.9763	−0.4085	0.087*	
C5	−0.1598 (4)	0.8419 (3)	−0.31035 (18)	0.0753 (6)	
H5	−0.2979	0.8154	−0.3636	0.09*	
C6	−0.1672 (3)	0.7846 (3)	−0.20373 (16)	0.0615 (5)	
H6	−0.3114	0.7203	−0.1851	0.074*	
C7	0.0480 (3)	0.7661 (2)	−0.00927 (14)	0.0442 (4)	
O8	0.2338 (2)	0.80629 (17)	0.05733 (11)	0.0633 (4)	
N9	−0.1594 (2)	0.66558 (19)	0.02071 (12)	0.0467 (4)	
H9	−0.284 (3)	0.634 (2)	−0.0281 (16)	0.054 (5)*	
C10	−0.1998 (3)	0.6059 (2)	0.12532 (14)	0.0446 (4)	
O11	−0.3996 (2)	0.52249 (17)	0.13780 (10)	0.0603 (4)	
N12	−0.0057 (2)	0.64570 (19)	0.20766 (11)	0.0467 (4)	
H12	0.131 (4)	0.707 (2)	0.1906 (17)	0.067 (6)*	
C13	0.0039 (3)	0.6057 (2)	0.31929 (13)	0.0447 (4)	
S14	0.27039 (8)	0.66392 (7)	0.40142 (4)	0.05930 (19)	
N15	−0.1971 (3)	0.5267 (3)	0.35341 (15)	0.0646 (5)	
H15A	−0.336 (4)	0.507 (2)	0.3052 (18)	0.075 (6)*	
H15B	−0.199 (3)	0.486 (2)	0.4173 (18)	0.066 (6)*	

### Atomic displacement parameters (Å^2^)

**Table:**

	*U*^11^	*U*^22^	*U*^33^	*U*^12^	*U*^13^	*U*^23^
C1	0.0468 (9)	0.0459 (10)	0.0402 (9)	0.0048 (8)	0.0046 (7)	0.0100 (8)
C2	0.0539 (10)	0.0726 (13)	0.0505 (11)	0.0013 (9)	0.0049 (8)	0.0214 (10)
C3	0.0714 (13)	0.0858 (16)	0.0630 (14)	0.0016 (12)	0.0175 (11)	0.0337 (12)
C4	0.0944 (16)	0.0782 (15)	0.0503 (12)	0.0174 (13)	0.0138 (12)	0.0294 (11)
C5	0.0810 (15)	0.0890 (16)	0.0523 (12)	0.0069 (13)	−0.0107 (11)	0.0263 (11)
C6	0.0564 (11)	0.0714 (13)	0.0518 (12)	−0.0029 (9)	−0.0038 (9)	0.0230 (10)
C7	0.0403 (9)	0.0483 (10)	0.0410 (9)	0.0009 (7)	0.0013 (7)	0.0102 (8)
O8	0.0426 (6)	0.0882 (10)	0.0501 (7)	−0.0140 (6)	−0.0047 (6)	0.0269 (7)
N9	0.0392 (7)	0.0609 (10)	0.0353 (8)	−0.0021 (7)	−0.0023 (6)	0.0141 (7)
C10	0.0411 (9)	0.0546 (11)	0.0349 (9)	0.0016 (8)	0.0012 (7)	0.0099 (8)
O11	0.0397 (6)	0.0898 (10)	0.0432 (7)	−0.0107 (6)	−0.0017 (5)	0.0213 (6)
N12	0.0370 (7)	0.0637 (10)	0.0359 (8)	−0.0022 (7)	0.0008 (6)	0.0165 (7)
C13	0.0401 (8)	0.0588 (11)	0.0355 (9)	0.0069 (8)	0.0047 (7)	0.0129 (8)
S14	0.0402 (2)	0.0911 (4)	0.0424 (3)	0.0002 (2)	−0.00342 (18)	0.0213 (2)
N15	0.0403 (8)	0.1120 (15)	0.0414 (9)	0.0026 (9)	0.0021 (7)	0.0336 (10)

### Geometric parameters (Å, º)

**Table:**

C1—C6	1.377 (2)	C7—O8	1.2181 (18)
C1—C2	1.383 (2)	C7—N9	1.378 (2)
C1—C7	1.483 (2)	N9—C10	1.391 (2)
C2—C3	1.376 (3)	N9—H9	0.843 (18)
C2—H2	0.93	C10—O11	1.2205 (18)
C3—C4	1.353 (3)	C10—N12	1.3591 (19)
C3—H3	0.93	N12—C13	1.387 (2)
C4—C5	1.377 (3)	N12—H12	0.882 (19)
C4—H4	0.93	C13—N15	1.305 (2)
C5—C6	1.385 (3)	C13—S14	1.6679 (16)
C5—H5	0.93	N15—H15A	0.91 (2)
C6—H6	0.93	N15—H15B	0.85 (2)
			
C6—C1—C2	118.74 (16)	O8—C7—N9	120.79 (15)
C6—C1—C7	124.45 (15)	O8—C7—C1	121.33 (14)
C2—C1—C7	116.81 (15)	N9—C7—C1	117.87 (14)
C3—C2—C1	120.69 (18)	C7—N9—C10	128.80 (14)
C3—C2—H2	119.7	C7—N9—H9	119.4 (12)
C1—C2—H2	119.7	C10—N9—H9	111.8 (12)
C4—C3—C2	120.02 (18)	O11—C10—N12	124.46 (15)
C4—C3—H3	120	O11—C10—N9	119.83 (14)
C2—C3—H3	120	N12—C10—N9	115.71 (14)
C3—C4—C5	120.70 (19)	C10—N12—C13	127.81 (14)
C3—C4—H4	119.7	C10—N12—H12	117.8 (13)
C5—C4—H4	119.7	C13—N12—H12	114.4 (13)
C4—C5—C6	119.38 (19)	N15—C13—N12	117.68 (14)
C4—C5—H5	120.3	N15—C13—S14	124.41 (13)
C6—C5—H5	120.3	N12—C13—S14	117.91 (12)
C1—C6—C5	120.46 (18)	C13—N15—H15A	118.8 (13)
C1—C6—H6	119.8	C13—N15—H15B	121.9 (13)
C5—C6—H6	119.8	H15A—N15—H15B	119.1 (18)

### Hydrogen-bond geometry (Å, º)

**Table:**

*D*—H···*A*	*D*—H	H···*A*	*D*···*A*	*D*—H···*A*
N9—H9···O11^i^	0.843 (18)	2.172 (18)	2.9891 (18)	163.3 (16)
N12—H12···O8	0.882 (19)	1.919 (19)	2.6171 (17)	135.0 (17)
N15—H15*A*···O11	0.91 (2)	1.99 (2)	2.684 (2)	132.0 (17)
N15—H15*B*···S14^ii^	0.85 (2)	2.58 (2)	3.4295 (17)	171.3 (17)

Symmetry codes: (i) −*x*−1, −*y*+1, −*z*; (ii) −*x*, −*y*+1, −*z*+1.
